# Development and Evaluation of Nystatin-Loaded Novasomal Gel for the Treatment of *Candida albicans* Infection: In Vitro Microbiological and Skin Compatibility Study

**DOI:** 10.3390/gels11100774

**Published:** 2025-09-25

**Authors:** Muhammad Abid Mustafa, Muhammad Fahad, Maryam Mughal, Namra Rasheed, Saad S. Alqahtani, Muhammad Zahid Iqbal

**Affiliations:** 1 Department of Pharmaceutics, Faculty of Pharmaceutical Sciences, Lahore University of Biological and Applied Sciences, Lahore 53400, Pakistan; pogords9@gmail.com (M.F.); maryamblacksmith@gmail.com (M.M.); namrarasheed8@gmail.com (N.R.); 2 Department of Clinical Pharmacy, College of Pharmacy, King Khalid University, Abha 61421, Saudi Arabia; ss.alqahtani@kku.edu.sa (S.S.A.); mzmuhammad@kku.edu.sa (M.Z.I.)

**Keywords:** nystatin, transdermal drug delivery, fungal infections, ethanol injection, vesicular system, skin compatibility

## Abstract

Candida infections pose a significant health threat, and conventional antifungal drugs like nystatin are limited due to poor solubility, skin permeability, and frequent dosage requirements. Nystatin effectively targets Candida species by disrupting cell membranes, but formulation issues hinder clinical use. Lipid-based vesicular carriers, or novasomes, provide controlled, prolonged drug release and enhanced skin penetration. This study focuses on developing nystatin-loaded novasomal gels as an advanced drug delivery system to enhance therapeutic efficacy, bioavailability, and patient compliance. The formulation was prepared using a modified ethanol injection technique, combining stearic acid, oleic acid, Span 60, cholesterol, and Carbopol to produce a stable transdermal gel. Comprehensive in vitro characterization using FTIR, SEM, XRD, and thermal analysis confirmed the chemical compatibility, morphological uniformity, and physical stability of the nystatin-loaded novasomal gel. Entrapment efficiency differed significantly among the formulations (*p* < 0.05), with F7 achieving the highest value (80%). All formulations maintained pH levels within the skin-friendly range of 5.5 to 7.0. Viscosity measurements, ranging from 3900 ± 110 to 4510 ± 105 cP, confirmed their appropriate consistency for dermal use. Rheological analysis showed a dominant elastic response, as indicated by storage modulus values consistently higher than the loss modulus. Particle size ranged from 4143 to 9570 nm, while PDI values remained below 0.3, reflecting uniform particle distribution. Zeta potential values were strongly negative, supporting physical stability. XRD studies indicated reduced crystallinity of nystatin within the formulations, while FTIR confirmed drug-excipient compatibility. SEM images showed spherical particles within the micrometer range. In vitro release studies demonstrated sustained drug release over 12 h, with F6 releasing the highest amount. The novasomal gel formulations-maintained stability for 30 days, with no notable alterations in pH, viscosity, or entrapment efficiency. Antifungal evaluation showed a larger inhibition zone (23 ± 2 mm) compared with the plain drug solution (15 ± 1.6 mm), while the MIC value was reduced (4.57 µg/mL), indicating greater potency. Skin irritation assessment in rats revealed only minor, temporary erythema, and the calculated Primary Irritation Index (0.22) confirmed a non-irritant profile. These findings suggest that the developed novasomal gel offers a promising approach for enhancing the treatment of fungal infections by enabling prolonged drug release, minimizing dosing frequency, and improving patient compliance.

## 1. Introduction

Fungal infections, especially those caused by Candida species, are increasingly prevalent in both healthcare environments and the general population. These infections can affect various parts of the body, including the skin, mucous membranes, and internal organs [1]. A 2024 review found that invasive fungal infections affect over 6.5 million people annually, causing 3.8 million deaths. Major causes include aspergillosis, candidiasis, pneumonia, and cryptococcal meningitis. Atopic fungal asthma affects 11.5 million people annually and leads to 46,000 deaths. In 2021, 5.62 million pulmonary fungal infections caused 45,500 deaths, with increasing impacts in low- and middle-income countries and older age groups [2].

Nystatin is a polyene antifungal drug that targets both pathogenic and non-pathogenic yeasts and fungi, primarily targeting Aspergillus and Candida species. It attaches to ergosterol in the fungal cell membrane, disrupting its integrity and creating holes for essential ions like potassium to leak out. This makes nystatin an effective treatment for various fungal infections, as it causes the cell to lose its membrane potential, become acidic, and ultimately die [3]. Nystatin is a polyene macrolide antifungal (C_47_H_75_NO_17_, MW ~ 926 g/mol), structurally analogous to amphotericin B. 

**Figure 1:**
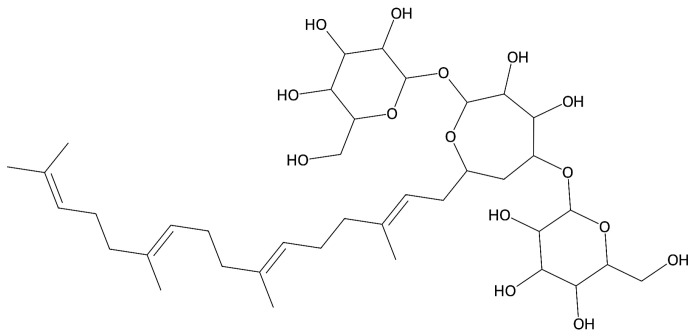
Chemical structure of nystatin.

The presence of conjugated double bonds and functional groups defines its unique physicochemical profile. To complement these features, the chemical structure of nystatin is presented in Section 1 (Figure 1), providing a clearer representation of the molecular framework discussed. It is practically insoluble in water and many organic solvents, though somewhat more soluble in methanol (~11.2 mg/mL), ethylene glycol (~8.8 mg/mL), and DMSO (~5 mg/mL). Quantitative data support low aqueous solubility (~0.066 g/L) and a modest lipophilicity (log P ≈ 0.5). Due to negligible gastrointestinal and topical absorption, nystatin remains limited to treating localized superficial and GI Candida infections, with minimal systemic exposure or metabolism. Because of these limitations, traditional formulation techniques are less successful, particularly when it comes to mucosal and transdermal delivery [4]. To address these challenges, there has been a growing interest in advanced drug delivery systems designed to enhance the solubility, stability, and bioavailability of drugs, particularly those intended for topical use. New drug delivery systems like liposomes, solid lipid nanoparticles, and nanoemulsions have been developed to address these issues, but issues like drug leakage, physical instability, and inadequate skin penetration persist [5].

Novasomes, a subclass of vesicular carriers with a multi-lamellar vesicle system and higher internal phase volume, offer a promising alternative. Their biphasic nature enables simultaneous encapsulation of both water-soluble and hydrophilic drugs, improving solubility. These lipid vesicles help stabilize the drug, protect against deterioration, and improve skin penetration. By effectively delivering drugs directly to the infection site, novasomes minimize systemic side effects and the need for frequent applications [6].

Novasomes, when incorporated into a gel matrix, enhance drug retention, prolong release, and reduce systemic side effects. It also serves as a mucoadhesive reservoir, improving penetration and uptake at the target site [7]. Given the promising results of clinical trials involving various drugs, novasomal transdermal gels are positioned as a potential innovation in drug delivery systems. A promising treatment for fungal infections is the incorporation of nystatin into novasomal carriers and its formulation into a gel for transdermal administration. The gel base simplifies application and improves skin adherence, ensuring a gradual, regulated release of the drugs [8].

This study aims to develop and characterize a nystatin-loaded novasomal gel for enhanced topical treatment of fungal infections. The primary objective is to improve the drug’s skin permeability, therapeutic efficacy, and patient compliance by incorporating it into a stable, biocompatible transdermal delivery system. Comprehensive in vitro evaluations were conducted to assess the formulation’s physicochemical properties, antifungal activity, release behavior, and dermatological safety. By leveraging the advantages of novasomal technology, this research seeks to contribute to the advancement of topical antifungal therapies and the broader field of vesicular drug delivery systems.

## 2. Results and Discussion

### 2.1. Results

#### 2.1.1. Formulation Physical Appearance

The prepared formulations exhibited distinct physical appearances. The unloaded novasomes appeared as a milky, opaque suspension, whereas the nystatin-loaded novasomes showed a yellow to tanned coloration with a similar turbid and opaque consistency. Before incorporation into the gel base, the formulations were free-flowing liquids, which transformed into gel-like systems upon incorporation of the gelling agent. All preparations were homogeneous in appearance with a smooth texture and showed no signs of visible aggregation or precipitation.

#### 2.1.2. Entrapment Efficiency and pH Measurement

The entrapment efficiency of the nine formulations varied significantly (*p* < 0.05) according to a one-way ANOVA. With an entrapment efficiency of 80%, F7 outperformed F2 (70%) and F4 (69%), which was significantly higher (*p* < 0.01). Likewise, the pH values were within the physiologically acceptable range of 5.5 to 7.0 for topical application, with F6 and F7 exhibiting the highest pH values (6.99 and 6.98, respectively). Statistical analysis (*p* < 0.05) indicated significant variations in pH among the formulations; however, all values remained within the safe range for skin application, ensuring the suitability of the prepared formulations. The results are shown in Table 1.

#### 2.1.3. Viscosity and Rheological Properties

Table 1 provides a summary of the rheological and viscosity profiles of the nine novasomal gel formulations. Viscosity values showed a suitable consistency for topical applications, ranging from 3900 ± 110 to 4510 ± 105 cP. F7 had the highest viscosity of all the formulations (4510 ± 105 cP). While the loss modulus (G″), which reflects viscous properties, ranged from 28 ± 2.0 to 39 ± 2.2 Pa, the storage modulus (G′), which indicates elastic behavior, varied between 172 ± 7 and 210 ± 9 Pa. The gels’ dominant elastic behavior was validated by the higher G′ than G″ in all formulations, indicating sufficient structural integrity for extended application.

#### 2.1.4. Zeta Potential, PDI, and Particle Size

The particle size, polydispersity index (PDI), and zeta potential of the nine novasomal formulations (F1–F9) were analyzed. The particle size values showed considerable variation, ranging from 4143 ± 37.8 nm for F7 to 9570 ± 41.2 nm for F2. This variation reflects the influence of formulation composition on vesicle size. PDI values ranged between 0.01 ± 0.006 and 0.29 ± 0.010, with most formulations showing values below 0.3, indicating an acceptable distribution of particle sizes. Notably, formulations F5 to F9 had PDI values below 0.1, suggesting a more uniform size distribution. Zeta potential measurements for all formulations were highly negative, with values ranging from −35.50 ± 5.0 mV to −79.10 ± 4.8 mV, indicating good surface charge stability and low risk of particle aggregation. Formulations with both high negative zeta potential and low PDI (particularly F5 to F9) demonstrated the most favourable physicochemical characteristics for stable delivery systems. The results are shown in Table 2.

#### 2.1.5. Thermal Analysis

**Figure 2:**
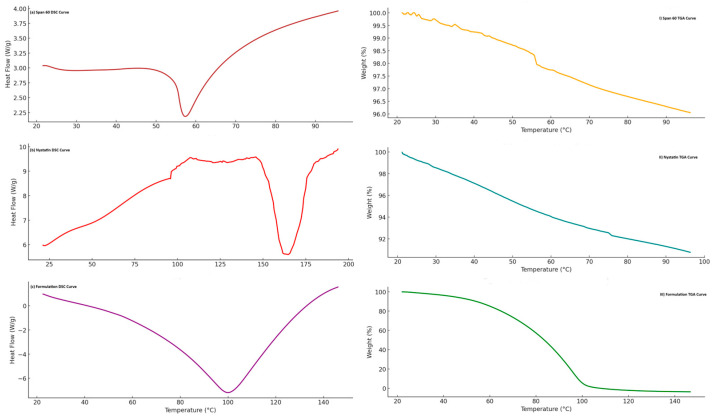
Differential scanning calorimetry (**a**) nystatin, (**b**) Span 60, and (**c**) formulation F6, and thermogravimetric analysis of (I) nystatin, (II) Span 60, and (III) formulation F6.

DSC analysis of Nystatin exhibited a distinct endothermic peak at 166.73 °C, corresponding to its melting point, as illustrated in Figure 2b. The TGA curve indicates that Nystatin starts to degrade at 30 °C and is completely degraded at 97 °C, as indicated in Figure 2(II).

DSC analyses of Span 60, an emulsifier, revealed an endothermic peak at 57 °C, indicating its transition from a solid to a liquid state as shown in Figure 2a. The TGA results revealed stability up to 55 °C, with degradation completed at 97 °C. These findings are shown in Figure 2(I).

The DSC thermograms of formulation F6, which contains cholesterol, stearic acid, Span 60, and nystatin, show a phase transition at 100 °C, indicating the loss of nystatin crystallinity and its dispersion in the novasomes, as illustrated in Figure 2c. The formulation F6 is stable up to 58 °C; however, TGA analysis reveals degradation beginning at 60 °C and continuing until 105 °C, suggesting mass loss. The results are shown in Figure 2(III).

#### 2.1.6. FTIR

**Figure 3:**
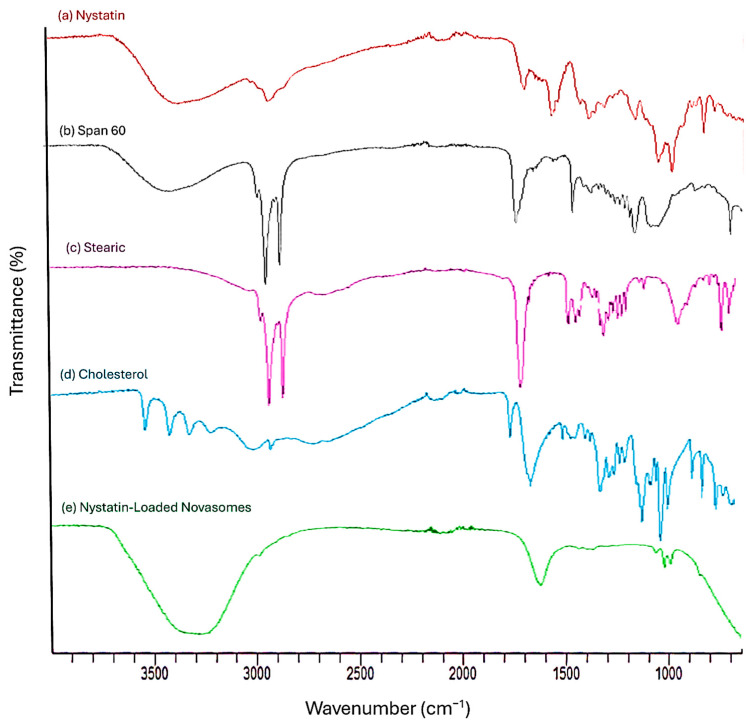
FTIR Spectra of (**a**) nystatin, (**b**) Span 60, (**c**) stearic acid, (**d**) cholesterol, and (**e**) nystatin-loaded novasomes (formulation F6).

FTIR peaks of nystatin are a band at approximately 1038 cm^−1^, which is attributed to polyene sequences. Additional distinctive peaks can be observed at 3314 cm^−1^ due to O–H stretching, 1600 cm^−1^ due to C=O stretching, 1416 cm^−1^ due to COOH bending, and 1029 cm^−1^ due to C–O–C stretching. Span 60 FTIR displays multiple significant FTIR peaks in the 2800–3000 cm^−1^ range because of C–H stretching, as well as a prominent peak at about 1738 cm^−1^ because of C=O stretching. FTIR analysis of stearic acid shows asymmetrical and symmetrical stretching of the –CH_2_ bonds, which are responsible for the primary bands, which are located at 2919 cm^−1^ and 2848 cm^−1^. The presence of the –COOH group is indicated by a peak at 1701 cm^−1^, while the asymmetric and symmetric stretches of the –COO– group may be connected to a peak at 1586.43 cm^−1^. FTIR analysis of cholesterol reveals key peaks at around 3400 cm^−1^, and the OH stretching vibration appears as a broad, intense band. In contrast, CH stretching vibrations, both symmetric and asymmetric, lie between 2800 and 3000 cm^−1^. Peaks include one at 1674 cm^−1^ for the C=C double bond in the second ring and another at 2899 cm^−1^ because of CH_2_ symmetric stretching. The FTIR spectrum of a formulation F6 includes broad peaks around 3200–3600 cm^−1^ for hydroxyl (O–H) groups and peaks between the smooth region of 2800–3000 cm^−1^, indicating the suppression of the C–H band, which can probably be explained by dense packing of the excipients and nystatin within novasomes and favorable excipient interactions leading to novasomes formation. These spectra are shown in Figure 3.

#### 2.1.7. SEM

**Figure 4:**
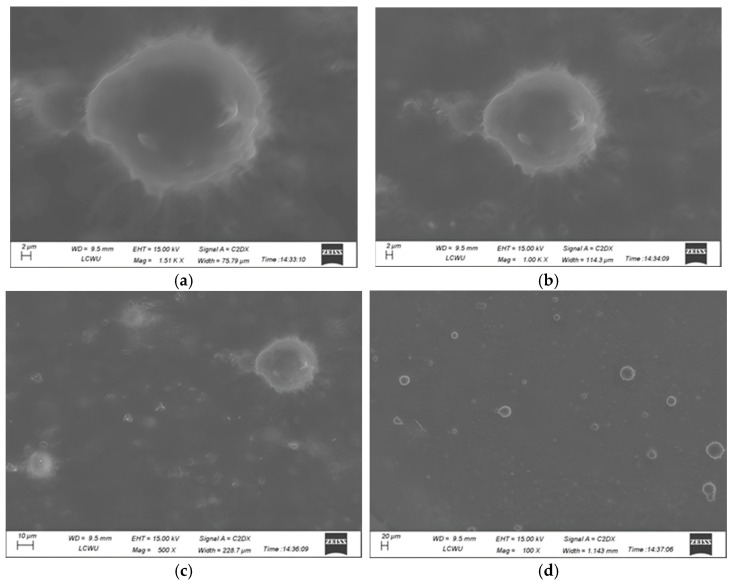
SEM micrographs of the formulation at different magnifications: (**a**) 1.51k×, (**b**) 1.0k×, (**c**) 500×, and (**d**) 100×.

The SEM images of formulation F6 (Figure 4a at 1.51k× and Figure 4c at 100×) and F8 (Figure 4b at 1.0k× and Figure 4d at 500×) reveal particles with sizes ranging from 2 µm to 20 µm. This size distribution falls within the desired specifications for optimal performance, as illustrated in Figure 4.

#### 2.1.8. XRD

**Figure 5:**
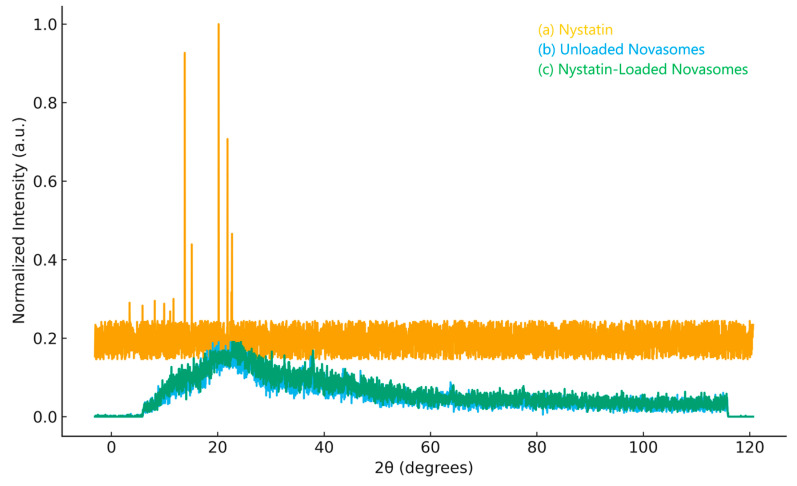
The XRD diffractogram: (**a**) nystatin, (**b**) unloaded novasomes, and (**c**) nystatin-loaded novasomes.

The XRD profile of pure nystatin (Figure 5a) revealed several distinct and intense diffraction peaks at 2θ values of 13.80° (380 counts), 15.13° (180 counts), 20.17° (410 counts), 21.85° (290 counts), 22.58° (130 counts), and 22.70° (191 counts). The presence of these sharp reflections confirms that the drug exists in a crystalline state, which is consistent with previously reported data for crystalline nystatin.

In comparison, the unloaded novasomes (Figure 5b) exhibited a broad diffuse halo with no prominent peaks. This pattern is typical of amorphous materials, indicating that the lipid and surfactant components are arranged in a disordered, non-crystalline manner.

Interestingly, once nystatin was incorporated into the novasomal system (Figure 5c), its characteristic crystalline reflections disappeared. The diffractogram closely resembled that of the unloaded carrier, dominated by a broad hump rather than sharp peaks. This finding suggests that the drug lost its long-range crystalline order during encapsulation and became dispersed within the lipid–surfactant matrix in a more amorphous state.

Such a reduction in crystallinity is commonly reported when drugs are incorporated into lipid-based carriers, as the molecular arrangement of the drug is disrupted by the surrounding matrix. This transformation from crystalline to amorphous form is advantageous, as it often improves drug solubility and may enhance bioavailability. Overall, the XRD results confirm the successful loading of nystatin into the novasomes.

#### 2.1.9. Stability Studies

The storage stability of novasomal transdermal gels was evaluated over a specified period under different conditions. The formulations were assessed for changes in physical appearance, pH, viscosity, and entrapment efficiency. In Table 3, the results indicated that the gels remained stable, with no significant changes in texture, phase separation, or precipitation. No statistically significant change was observed in pH, viscosity, or entrapment efficiency (*p* > 0.05). No visible signs of phase separation, colour change, or precipitation were observed throughout the 30 days under both storage conditions.

#### 2.1.10. Standard Calibration Curve

The drug was quantitatively analyzed using a validated UV spectrophotometric method. The standard calibration curve was linear across the concentration range of 2–12 µg/mL with an R^2^ of 0.9999 (see Supplementary Figure S1). Method validation parameters, including LOD, LOQ, accuracy, and precision, complied with ICH requirements and are summarized in Supplementary Table S1.

#### 2.1.11. In Vitro Drug Release

**Figure 6:**
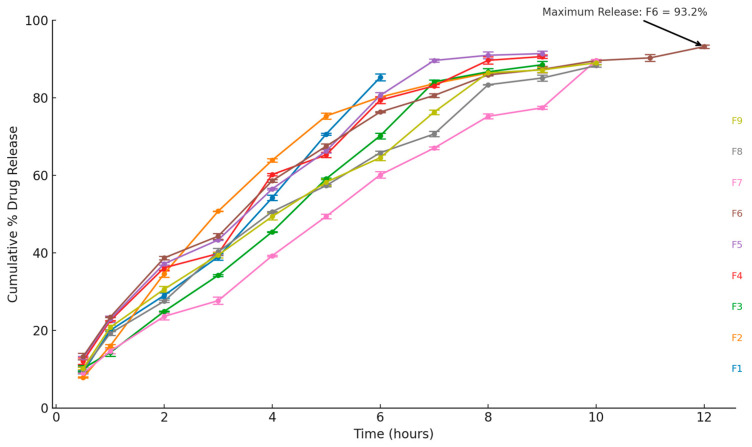
In vitro drug release profiles showing the percentage of drug released from all formulations over time.

The release kinetics of the novasome-based transdermal gel containing nystatin were evaluated. In vitro dissolution studies demonstrated a sustained drug release for up to 12 h across formulations F1 to F9, as shown in Figure 6. Most formulations achieved 80% of the drug release within 10 h, except for F7. The profiles suggest that these formulations can effectively sustain drug release over prolonged periods. Statistical analysis, summarized in Supplementary Table S2, confirmed that F6’s release was significantly higher compared to F1, F3, and F7 at each time point beyond 4 h (*p* < 0.05, one-way ANOVA).

#### 2.1.12. Release Kinetics Studies

The release kinetic studies were conducted using various kinetic models, including zero-order, first-order, Higuchi, and Korsmeyer–Peppas, to evaluate the drug release pattern. The R^2^ value of the zero-order model is closer to 1 compared to the first-order model. The *n* values ranged from 0.73 to 0.89, indicating a non-Fickian release mechanism. Additionally, the Higuchi model exhibited values close to 1 with minimal deviation. A comprehensive summary of the findings is presented in Table 4.

#### 2.1.13. Antifungal Activity of Nystatin-Loaded Novasomes

(a) Agar wall diffusion method

**Figure 7:**
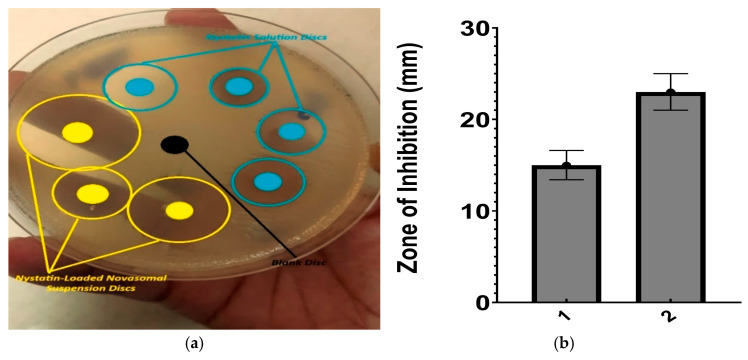
(**a**) Antifungal activity of blank disc, nystatin solution disc, and nystatin-loaded novasomal disc; (**b**) 1. zone of inhibition of nystatin and 2. nystatin-loaded novasomal gel.

The zone of inhibition for the nystatin solution was 15 ± 1.6 mm, whereas the nystatin-loaded formulation demonstrated a significantly larger zone of 23 ± 2 mm, indicating enhanced antifungal activity. Figure 7a illustrates the improved antifungal effect, while Figure 7b displays the corresponding zone of inhibition.

(b) Minimum inhibitory concentration

The MIC was found to be 4.57 µg/mL for the nystatin-unloaded novasomes and 6.98 µg/mL for nystatin using the checkerboard assay.

#### 2.1.14. In Vivo Skin Irritation

**Figure 8:**
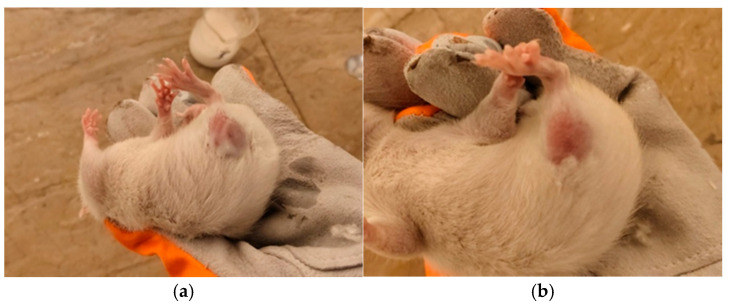
(**a**) Novasomal gel application; (**b**) after novasomal gel application.

The potential of test formulation 6 to cause skin irritation was assessed in adult rats using a Draize-based procedure. Following topical application, erythema and edema were scored at 24, 48, and 72 h (scale 0–4). Individual animal data are provided in Table 5. A single rat (R1) showed mild, transient erythema (score 1) at 24 and 48 h, while no edema was observed in any animal throughout the study period. At 72 h, all animals were free of visible reactions. The mean combined irritation scores (erythema + edema) were 0.33 ± 0.58 at 24 and 48 h, and 0.00 ± 0.00 at 72 h (*n* = 3). The calculated Primary Irritation Index (PII) was 0.22, which is below the threshold of 0.5 and therefore classifies the formulation as non-irritant to rat skin under the experimental conditions. There is no sign of any reaction after 72 h shown in Figure 8.

### 2.2. Discussion

The nystatin-loaded novasomal gel aims to improve nystatin’s therapeutic efficacy in treating fungal infections by improving stability, bioavailability, and controlled release. This lipid-based vesicular system addresses challenges such as poor skin penetration and inconsistent drug release. Microbial and skin compatibility assessment confirms its potential as an advanced transdermal treatment, providing prolonged therapeutic action and improved effectiveness against fungal infections.

The formulations showed distinct and pharmaceutically acceptable physical characteristics. Unloaded novasomes appeared milky and opaque, while nystatin-loaded systems exhibited a yellow to tanned hue, consistent with the color imparted by polyene antifungals. All preparations were initially free-flowing liquids that converted into smooth, homogeneous gels without aggregation upon addition of the gelling agent. These findings align with the expected behavior of lipid-based vesicular carriers and agree with previous reports that antifungal-loaded gels maintain homogeneity and acceptable appearance, supporting stability and patient compliance. These observations are consistent with previous reports; for example, Farooq et al. (2022) noted that voriconazole-loaded transethosomal gels also exhibited a smooth, homogeneous, milky appearance suitable for topical application [9].

The pH values of all formulations were within the physiologically acceptable range for topical use (5.5–7.0). Although F6 and F7 displayed slightly higher values (6.99 and 6.98), the differences across the formulations were not statistically significant. This consistency is important, as it ensures that the preparations are unlikely to cause skin irritation or discomfort during application. Comparable acceptable pH values have been reported by Imam et al. (2023), where a miconazole-loaded bilosome gel was found to have a pH of approximately 6.4 ± 0.1, supporting the skin-compatibility of vesicular gel formulations [10]. The entrapment efficiency results showed noticeable differences among the nine formulations, highlighting the effect of composition on drug loading capacity. Among them, F7 achieved the highest efficiency (80%), which was significantly greater than F2 (70%) and F4 (69%). A higher entrapment efficiency is generally desirable, as it ensures more drug is retained within the carrier, potentially improving therapeutic outcomes. Comparable entrapment efficiencies have been observed in the literature; Abdelbari et al. (2024) reported ~75% EE for their optimized novasome formulation, which is close to our highest efficiency (80% in F7), reinforcing that careful formulation design can yield high drug loading [11].

Rheological and viscosity measurements provided further insight into the suitability of the formulations as topical gels. Viscosity values ranged from 3900 to 4510 cP, with F7 again standing out as the most viscous. This thicker consistency may improve retention on the application site, supporting prolonged drug contact with the skin. Across all samples, the storage modulus (G′) exceeded the loss modulus (G″), confirming that the gels displayed predominantly elastic rather than viscous behavior. This balance suggests good structural integrity, which is favorable for maintaining stability and ease of application. Taken together, these findings identify F7 as the most promising formulation. Recent studies on miconazole nanogels reported viscosities of ~3200–4200 cP, similar to our values (3900–4510 cP), confirming that higher viscosity supports better skin retention. Like our gels, they also showed G′ > G″, indicating predominantly elastic behavior that ensures structural integrity and stability [12]. Its high entrapment efficiency, acceptable pH, greater viscosity, and dominant elastic behavior suggest it offers both stability and effectiveness for topical delivery.

The evaluation of particle size, PDI, and zeta potential highlighted clear differences among the nine novasome formulations (F1–F9). Particle sizes ranged widely, from about 4.1 μm in F7 to nearly 9.6 μm in F2, showing how strongly composition can affect vesicle dimensions. Despite this variation, most formulations maintained PDI values below 0.3, which indicates a reasonably uniform distribution of particle sizes. In fact, F5 through F9 stood out with PDIs below 0.1, suggesting particularly well-controlled and homogeneous vesicle populations. Zeta potential values for all formulations were highly negative, ranging between −35.50 and −79.10 mV. Such strong surface charges imply good electrostatic repulsion between particles, reducing the chance of aggregation and supporting overall stability. Taken together, the combination of low PDI and high negative zeta potential in F5–F9 points to these formulations as having the most favourable physicochemical properties for stable drug delivery. Similar outcomes were observed by Hani et al. (2024), who reported a nanosponge-based lumiracoxib gel with acceptable particle size, PDI, and zeta potential [13].

The thermal studies gave us valuable insights into how stable our materials are under heat. DSC analysis of Nystatin showed a significant thermal change at 166.73 °C, corresponding to its melting point. The TGA results showed it starts breaking down at around 30 °C and is fully degraded by 97 °C, meaning it needs to be stored at lower temperatures to stay effective. Span 60, our emulsifier, melts at around 57 °C and remains stable up to 55 °C before it starts to degrade. Interestingly, when we combined Nystatin, Span 60, cholesterol, and stearic acid into our novasomal formulation, we saw improved stability. The DSC showed an apparent phase change at 100 °C, pointing to good dispersion of nystatin in the system. The formulation held up well until about 58 °C, with degradation starting slowly after that. Overall, putting these ingredients into a single formulation made them more thermally stable, which is great news for shelf life and storage. It shows how the formulation helps protect the active ingredients, enhancing its reliability for future use. In their 2016 study, ‘Trans-nasal zolmitriptan novasomes: in vitro preparation, optimization, and in vivo evaluation of brain targeting efficiency’, Radwa M. A. Abd-Elal et al. found a similar result [14].

The presence and compatibility of nystatin and the excipients in the formulation have been confirmed through FTIR analysis. Nystatin exhibited three prominent peaks: a broad O–H stretch at 3314 cm^−1^, a carbonyl (C=O) stretch near 1600 cm^−1^, and another at 1038 cm^−1^ associated with its polyene structure. Span 60 showed a strong ester C=O peak at 1738 cm^−1^, along with typical C–H stretching between 2800 and 3000 cm^−1^. Stearic acid also displayed distinct signals for the COOH and COO^−^ groups at 1701 and 1586 cm^−1^, and it featured CH_2_ stretching peaks at 2919 and 2848 cm^−1^. Cholesterol presented a broad O–H peak around 3400 cm^−1^, followed by a C=C stretch at 1674 cm^−1^ and a CH stretch between 2800 and 3000 cm^−1^. In the final formulation, all these characteristic peaks were present—broad O–H peaks (3200–3600 cm^−1^), aliphatic C–H bands (2800–3000 cm^−1^), indicating the suppression of the C–H band, which probably can be explained by dense packing of the excipients and nystatin within novasomes and favourable excipients interactions leading to novasome formation. Iman Fatima et al. noticed a similar pattern in their 2022 study, ‘Novasomes as Nano-Vesicular Carriers to Enhance Topical Delivery of Fluconazole: A Novel Approach to Treat Fungal Infections’ [15].

SEM analysis confirmed the formulations’ smooth surface morphology and uniform particle size distribution, with sizes ranging from 2 µm to 20 µm. Its distinctive vesicular structure makes it suitable for transdermal delivery, suggesting enhanced drug encapsulation and increased bioavailability. XRD analysis showed that pure nystatin had sharp peaks, confirming its crystalline nature. In contrast, the unloaded novasomes displayed a broad halo, typical of an amorphous structure. Once nystatin was incorporated, its crystalline peaks disappeared, and the pattern resembled that of the carrier. This indicates that the drug became molecularly dispersed within the lipid–surfactant matrix and shifted into an amorphous form. Such a transition is important, as amorphous drugs generally dissolve better than their crystalline counterparts, which may enhance solubility and bioavailability. These findings confirm successful loading of nystatin into the novasomes and suggest a potential advantage for drug delivery. Similar to the observations of Benavent et al. (2021), where nystatin solid dispersions showed amorphization in XRPD, our XRD results also revealed that nystatin-loaded novasomes lost crystalline peaks and became molecularly dispersed, suggesting improved solubility [16].

The novasomal gels remained stable for 30 days at both 25 °C and 4 °C, showing no phase separation, precipitation, or color change. pH (6.83–6.89), viscosity (4295–4325 cP), and entrapment efficiency (79.1–80.9%) exhibited no significant variation (*p* > 0.05). These results confirm short-term stability and support their reliability for transdermal delivery. Comparable outcomes were reported by Jacob et al. (2025) [17], where a tadalafil nanoemulgel retained stability, acceptable pH, and appropriate gel consistency while providing sustained release. In line with this, our novasomal gels remained stable for 30 days with consistent physicochemical properties, confirming their suitability for transdermal application [17].

Studies on the in vitro release of nystatin from novasomal transdermal gels demonstrated a controlled release profile over 12 h, with most formulations achieving 80% drug release by 10 h, except for formulation F7. The initial release phase was slower, followed by a stable release pattern, indicating that the novasomal system effectively regulates drug release due to its vesicular structure, which modulates the diffusion of nystatin. These results confirm that the gel platform is suitable for extended and controlled transdermal delivery of antifungal agents like nystatin.

Multiple kinetic models were employed to analyze the study of nystatin-loaded novasomal transdermal gels to understand the drug release mechanism. The results demonstrated the formulations’ controlled and concentration-independent drug release over time, which is ideal for long-term transdermal therapy. The Korsmeyer–Peppas model indicated a non-Fickian transport mechanism, suggesting a combination of drug diffusion and polymer matrix relaxation, while the Higuchi model proposed diffusion through the gel matrix. By effectively sustaining a regulated and prolonged nystatin release, the novasomal gel system enhances its potential for effective transdermal drug administration and boosts patient compliance. A similar pattern was reported by Sadek Ahmed et al. during the investigation of exploring the potential of antifungal-loaded proniosomes to consolidate corneal permeation in fungal keratitis: A comprehensive investigation from laboratory characterization to microbiological evaluation in 2025 [18].

Nystatin-loaded novasomal transdermal gels showed promising antifungal activity against *Candida albicans*, a common fungal pathogen. The enhanced therapeutic potential of the novasomal delivery system is likely due to the unique structural and physicochemical properties of novasomes, which facilitate better drug encapsulation, stability, and sustained release at the target site. The minimum inhibitory concentration (MIC) values were obtained via the checkerboard assay. The MIC for the nystatin-loaded novasomes was markedly lower (4.57 µg/mL) than that of the free nystatin solution (6.98 µg/mL), indicating improved therapeutic efficacy. As a fatty acid, stearic acid can disrupt fungal cell membranes, and oleic acid contains fixed-bend C=C bonds, allowing it to occupy a broader cross-section when entering the fungal membrane, thereby facilitating deeper penetration of the drug into the fungal cells. This synergistic effect between the delivery system and the active agent likely accounts for the improved efficacy of the formulation. This could offer an effective alternative to conventional topical formulations, particularly in managing resistant or recurrent fungal infections. Hamdy Abdelkader et al. reported a similar finding in their 2023 study, entitled ‘Formulation and optimization of lipid- and Poloxamer-tagged niosomes for dermal delivery of terbinafine: preparation, evaluation, and in vitro antifungal activity’ [19].

The Draize irritation test in rats showed that the formulation caused only minimal, transient erythema in a single animal, which resolved within 72 h. No edema was observed, and the Primary Irritation Index (PII) was 0.22, classifying the formulation as non-irritant. These results indicate good dermal tolerability and support the safe use of the formulation for topical application. Our Draize irritation results (PII = 0.22, non-irritant) are comparable to those reported for hydrolyzed keratin, which showed a similar PII value of 0.2 in rabbits, confirming minimal irritation and good dermal tolerability [20].

**Scheme 1:**
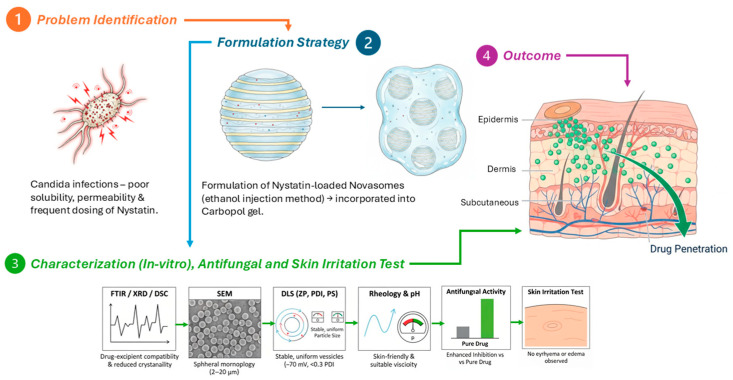
Schematic representation.

The study suggests novasome-based gels as a promising alternative to traditional topical antifungal treatments for fungal infections due to their controlled release, enhanced skin penetration, and improved therapeutic efficacy. Further clinical evaluations are needed to confirm its effectiveness and safety in humans, but the results provide a strong foundation for future transdermal drug delivery (Scheme 1).

## 3. Conclusions

This study developed a nystatin-loaded novasomal gel for treating fungal infections, overcoming challenges such as poor solubility and frequent dosing. The gel demonstrated enhanced antifungal activity, skin penetration, and sustained drug release. Its characterization confirmed successful nystatin encapsulation, stability, and good dermatological compatibility. This promising alternative offers a convenient and effective treatment, potentially improving patient compliance and reducing systemic side effects. However, the present work was limited to in vitro and preliminary evaluations; further in vivo studies, long-term stability assessments, and clinical investigations are needed to fully establish its therapeutic potential. Future research should also explore scale-up feasibility and patient acceptability to translate this formulation into clinical use.

## 4. Materials and Methods

### 4.1. Materials

For the development of the novasomal system, nystatin was employed as the model drug (Batch No. 0000420041; CAS No. 1400-61-9; Sigma-Aldrich, St. Louis, MO, USA). The formulation was composed of oleic acid (Batch No. SHBR0628; CAS No. 112-80-1; Merck, Darmstadt, Germany), stearic acid (Batch No. SHBS1369; CAS No. 57-11-4; Sigma-Aldrich, St. Louis, MO, USA), Span 60 (sorbitan monostearate, Batch No. 0000414007; CAS No. 1338-41-6; Sigma-Aldrich, St. Louis, MO, USA) as the surfactant, and cholesterol (Batch No. 0000479102; CAS No. 57-88-5; Sigma-Aldrich, St. Louis, MO, USA). Additional excipients included ethanol (CAS No. 64-17-5; Merck, Darmstadt, Germany) and Carbopol 940 (Carbomer, CAS No. 9003-01-4; Lubrizol, Wickliffe, OH, USA), which served as the gelling agent.

For antifungal evaluations, resazurin dye (Batch No. 0000321963; CAS No. 62758-13-8; Sigma-Aldrich, St. Louis, MO, USA) was used as a cell viability indicator, while dimethyl sulfoxide (CAS No. 67-68-5; Merck, Darmstadt, Germany) acted as the solvent. Culture media were prepared using Sabouraud dextrose agar (CM0041B Oxoid, Altrincham, Cheshire, UK). Phosphate buffer solutions were obtained with sodium hydroxide (CAS No. 1310-73-2; Merck, Darmstadt, Germany) and sodium dihydrogen phosphate (CAS No. 7558-80-7; Sigma-Aldrich, St. Louis, MO, USA).

All reagents and excipients were analytical grade and used without further modification. *Candida albicans* strain 144C (ATCC^®^ 10231™, American Type Culture Collection, Manassas, VA, USA) served as the fungal model for biological assays.

### 4.2. Preparation of Phosphate Buffer Solution (pH: 7.4)

A phosphate-buffered solution was prepared by dissolving 6.8 g of sodium dihydrogen phosphate in 1 L of distilled water under continuous stirring. The pH was adjusted to 7.4 by the gradual addition of sodium hydroxide solution, with constant monitoring using a calibrated pH meter.

### 4.3. Preparation of Novasomes and Novasomal Gel

Nystatin-loaded novasomes were developed following the ethanol injection method of Negi et al. [21], with a slight modification. An accurately weighed 10 mg of nystatin was dissolved in 5 mL of ethanol together with varying concentrations of oleic acid, stearic acid, span 60, and cholesterol under continuous stirring to ensure complete dissolution. The mixture was heated in a water bath at 60 °C to ensure complete dissolution of the material in ethanol. The ethanolic solution was then gradually injected into a five-fold larger volume of phosphate-buffered saline (PBS, pH 7.4) with continuous magnetic stirring at 500 rpm and 60 °C for 10 min, and the appearance of turbidity indicated the formation of novasomes. The dispersion was sonicated in a bath sonicator (Ultrasonic JPS-20A, Huanghua City, Cangzhou, Hebei, China) for 15 min at 25 ± 2 °C to achieve a uniform particle size. Finally, the prepared novasomal suspension was stored at 4 °C for further use. The accurate amount of Carbopol was added to the water and placed overnight to obtain a uniform carbopol gel. A 10 g quantity of Carbopol gel was taken, and then the novasomal suspension was gradually dispersed under continuous magnetic stirring at 500 rpm for 30 min to obtain a homogeneous novasomal gel formulation. A 30 g formulation of nystatin-loaded novasomal gel was successfully developed [21] (Table 6).

### 4.4. Evaluation of Nystatin-Loaded Novasomes Gel

#### 4.4.1. pH Measurements

A small sample of the gel was taken with a sterile spatula and placed in a beaker to measure the pH of the novasomal transdermal gel. The gel was then used to immerse a pH meter calibrated Hanna Instruments digital pH meter (Model: HI5221). Before measurement, the pH meter was calibrated using standard buffer solutions. To ensure homogeneity, the gel was gently stirred, and after the reading stabilized, the pH value was recorded. To ensure accuracy, this procedure was performed three times, and the average pH value was recorded [22].

#### 4.4.2. Viscosity and Rheological Evaluation

The viscosity of the prepared novasomal gels (F1–F9) was measured at 25 ± 1 °C and 50 rpm using a Brookfield DV2T digital viscometer equipped with spindle No. 4 [23]. Rheological behavior at 25 ± 1 °C was assessed using an Anton Paar MCR 102 rotational rheometer equipped with oscillatory sweep mode and a 25 mm parallel-plate geometry. The oscillatory frequency sweep mode was used to measure the storage modulus (G′) and loss modulus (G″) [24]. Each measurement was performed in triplicate, and the results are expressed using the mean ± standard deviation (SD).

#### 4.4.3. Entrapment Efficiency

Entrapment efficiency was determined indirectly by measuring the percentage of the free drug. The sample was subjected to cold centrifugation (CFGR-21SY, Infitek Inc., Lixia District, Jinan, China) at 4 °C for 1 h at 21,000 rpm. After centrifugation, the clear supernatant was carefully separated and diluted. The concentration of the unencapsulated drug was then quantified spectrophotometrically at 304 nm using a pre-established calibration curve. Entrapment efficiency was calculated using the following formula [25]:

$$\mathrm{EE}\;\%\;=\;\frac{Amount\;of\;Drug\;entrapped\;in\;vesicle-Amount\;of\;free\;drug\;in\;solution}{Total\;Amount\;of\;Drug}\;\times 100$$

#### 4.4.4. Zeta Potential, PDI, and Particle Size

The particle size (PS), polydispersity index (PDI), and zeta potential (ZP) of the prepared novasomes were measured using a Zetasizer (Malvern Panalytical Ltd., Malvern, UK) based on dynamic light scattering (DLS) and laser doppler micro-electrophoresis techniques [14].

#### 4.4.5. Thermal Analysis

The optimized formulation was initially frozen at −20 °C and then lyophilized for 24 h at −45 °C under reduced pressure using a freeze-dryer (BK-FD10PT, Biobase, High-tech Zone, Jinan, China). A dual DSC and TGA analyzer (Q600 TA instrument, New Castle, DE, USA) was utilized to evaluate the thermal properties. Each 2 mg sample was placed in an individual aluminum pan and heated to 350 °C in a nitrogen atmosphere. A scanning rate of 10 °C/min was employed to raise the temperature [26].

#### 4.4.6. Fourier Transform Infrared Spectroscopy (FTIR)

An FTIR (Cary-630, Agilent Technologies, Santa Clara, CA, USA) was utilized to record the FTIR spectra of the active ingredients nystatin, span 60, stearic acid, and cholesterol, as well as the optimized formulations that were lyophilized and loaded with or without drugs. The analysis spanned a spectral range of 4000–400 cm^−1^ and was performed at a controlled temperature of 25 ± 2 °C [27].

#### 4.4.7. Scanning Electron Microscopy (SEM)

The size and morphological properties of the optimized formulation were investigated using scanning electron microscopy (Evo LS10, Zeiss, Jena, Germany). A tiny droplet of the diluted sample was placed on a copper grid coated with carbon and allowed to dry at 25 ± 2 °C. The sample was stained with 2% (*w*/*v*) phosphotungstic acid to enhance contrast. SEM was used to capture images at the appropriate accelerating voltage [28].

#### 4.4.8. XRD Analysis

The nystatin, unloaded novasomal gel, and optimized nystatin-loaded novasomal formulation were subjected to lyophilization. Subsequently, Powder X-ray diffraction (XRD) analysis was performed using a D8 Discover diffractometer (Bruker, Karlsruhe, Germany) to evaluate the crystallinity of the nystatin, lyophilized unloaded formulation, and drug-loaded novasomes [14].

#### 4.4.9. Standard Curve for Drug Quantification

A standard calibration curve was established using nystatin dissolved in PBS with DMSO and measured at its λmax of 304 using a UV-Visible spectrophotometer (Shimadzu UV-1800, Tokyo, Japan).

#### 4.4.10. In Vitro Drug Release and Release Kinetics

The in vitro drug release of the novasomal transdermal gel was assessed using a Franz diffusion cell. The drug suspension was placed in the donor chamber, while the receptor compartment contained 15 mL of PBS (pH 7.4). The system was kept at 37 ± 0.5 °C with stirring for 12 h. A cellulosic dialysis membrane separated the donor and receptor compartments. At various time intervals (0.5, 1, 2, 4, 6, and up to 12 h), 1 mL samples were withdrawn, replaced with fresh PBS, filtered, and analyzed at 304 nm. The data of in vitro release were further evaluated for release kinetics using models such as Higuchi, zero order, first order, and Korsmeyer [29].

#### 4.4.11. Antifungal Activity of Nystatin-Loaded Novasomes

(a) Agar well diffusion method

A Sabouraud dextrose agar (SDA) was prepared and added to the Petri dish. The 10-fold diluted suspension of *Candida albicans* 144C (ATCC) was introduced into the Petri dish as a streak plate pattern. A sterile cork borer was used to create an 8 mm diameter disc containing 30 µL of nystatin solution to assess and compare efficacy through the disc diffusion method. Similarly, an optimized nystatin-loaded novasomal formulation was then introduced into the Petri dish, and the plate was incubated aerobically at 37 ± 2 °C for 48 h. The antifungal activity was recorded as the inhibition of visible microbial growth. The experiment was performed in triplicate for accuracy [30].

(b) Minimum Inhibitory Concentration (MIC)

To evaluate antimicrobial viability, the microdilution method was refined by incorporating resazurin dye as a redox indicator. *Candida albicans* 144C (ATCC) inoculates were prepared and adjusted to a concentration of 10^6^ CFU/mL. Novasomes loaded with nystatin, and nystatin alone, with a concentration of 32 µg/mL in DMSO (used as a reference drug), were each subjected to twofold serial dilutions in DMSO using a 96-well microplate. Each well was filled with 40 µL of brain–heart infusion broth as the growth medium, 10 µL of fungal inoculum, and a 50 µL test sample of Novasomes loaded with nystatin or nystatin alone [31]. The DMSO was used as the negative control. After 48 h of incubation at 37 °C ± 2 °C, 10 µL of resazurin dye was added to each well, which was then incubated for an additional hour in the dark at 37 °C. The colour changes, which signified microbial metabolic activity [32].

#### 4.4.12. In Vivo Skin Irritation

All procedures were approved by the Institutional Animal Care and Use Committee of Lahore University of Biological and Applied Sciences (Approval No: ERB-FOPS-DP/0133) and conducted in compliance with ethical standards and the 3Rs principle [33]. Twelve male Wistar rats (7–8 weeks, 180–220 g) were housed under controlled conditions (22 ± 2 °C, 55 ± 5% humidity, 12 h light/dark cycle) with free access to food and water. After one week of acclimatization, 1 g of nystatin-loaded novasomal gel was applied daily for seven days to a 4 cm^2^ shaved dorsal area. Skin irritation was assessed using a Draize-type protocol, scoring erythema and edema (0–4 scale) at 24, 48, and 72 h. The Primary Irritation Index (PII) was calculated from cumulative scores to evaluate dermal safety [34].

#### 4.4.13. Statistical Analysis

All experiments were carried out in triplicate, and the mean ± standard deviation (SD) is used to present the results. SPSS version 25 was used for statistical analysis. Multiple formulations were analyzed using one-way analysis of variance (ANOVA) to assess significant differences. *p*-values less than 0.05 were considered statistically significant.

## Figures and Tables

**Table 1:** Entrapment efficiency, pH, viscosity, and rheological properties of nystatin-loaded novasomal gels. Values are presented as mean ± SD (*n* = 3).

Formulation	Entrapment Efficiency (%)	pH (± SD)	Viscosity (cP)	Storage Modulus G′ (Pa)	Loss Modulus G″ (Pa)
F1	76.00 ± 1.20	5.71 ± 0.05	3900 ± 110	172 ± 7	28 ± 2.0
F2	70.00 ± 0.90	6.01 ± 0.04	4050 ± 120	180 ± 8	30 ± 2.2
F3	78.00 ± 1.10	6.36 ± 0.03	4150 ± 115	190 ± 8	31 ± 2.3
F4	69.00 ± 1.30	5.99 ± 0.02	4000 ± 108	178 ± 7	29 ± 2.1
F5	71.00 ± 1.00	6.65 ± 0.06	4200 ± 115	185 ± 8	32 ± 2.1
F6	79.00 ± 1.40	6.99 ± 0.02	4380 ± 98	198 ± 7	36 ± 2.4
F7	80.00 ± 1.50	6.98 ± 0.03	4510 ± 105	210 ± 9	39 ± 2.2
F8	74.00 ± 1.00	6.51 ± 0.04	4300 ± 112	193 ± 8	34 ± 2.3
F9	77.00 ± 1.20	6.59 ± 0.03	4400 ± 109	202 ± 8	35 ± 2.2

**Table 2:** Zeta potential, particle size, and polydispersity index of the prepared formulations. Values are presented as mean ± SD (*n* = 3).

Formulation	ZP (mV)	PS (nm)	PDI
F1	−35.50 ± 5.0	8836 ± 36.9	0.26 ± 0.015
F2	−39.14 ± 6.6	9570 ± 41.2	0.29 ± 0.010
F3	−51.72 ± 3.2	6492 ± 33.7	0.20 ± 0.012
F4	−45.35 ± 1.2	4991 ± 41.6	0.13 ± 0.009
F5	−79.10 ± 4.8	7734 ± 29.4	0.08 ± 0.005
F6	−75.93 ± 8.1	4725 ± 23.1	0.02 ± 0.003
F7	−70.44 ± 2.9	4143 ± 37.8	0.09 ± 0.007
F8	−77.26 ± 5.6	5139 ± 46.3	0.05 ± 0.002
F9	−67.48 ± 9.2	6122 ± 35.1	0.01 ± 0.006

Values expressed as mean ± SD, *n* = 3.

**Table 3:** Storage stability evaluation of novasomal gel formulations.

Parameter	Time Point	Room Temp (25 °C)	Refrigerated (4 °C)
pH	Day 0	6.85 ± 0.03	6.85 ± 0.05
	Day 15	6.83 ± 0.02	6.84 ± 0.09
	Day 30	6.89 ± 0.07	6.83 ± 0.01
Viscosity (cP)	Day 0	4320 ± 105	4320 ± 109
	Day 15	4310 ± 98	4325 ± 100
	Day 30	4295 ± 102	4312 ± 95
Entrapment Efficiency (%)	Day 0	80.7 ± 1.2	80.1 ± 1.7
	Day 15	79.1 ± 1.4	80.9 ± 1.3
	Day 30	79.4 ± 1.6	79.7 ± 1.5
Physical Appearance	Day 0	No change	No change
	Day 15	No change	No change
	Day 30	No change	No change

**Table 4:** Estimated kinetic parameters for drug release from nystatin-loaded novasomal gel.

Formulation	Zero-Order (K_0_, %·h^−1^)	R^2^	First-Order (K_1_, h^−1^)	R^2^	Higuchi (K_H, %·h^−1^/^2^)	R^2^	Korsmeyer– Peppas (K_KP, %·h^−n^)	*n*
F1	13.526	0.976	0.272	0.9437	30.821	0.6759	16.183	0.73
F2	13.995	0.9243	0.275	0.9587	29.251	0.8097	20.577	0.853
F3	12.857	0.9798	0.196	0.9845	31.253	0.7834	15.873	0.814
F4	12.451	0.9643	0.209	0.9476	27.36	0.8789	18.936	0.76
F5	13.321	0.9721	0.215	0.9572	28.014	0.6596	16.389	0.89
F6	9.177	0.9876	0.154	0.9618	24.876	0.8548	11.237	0.838
F7	8.451	0.9923	0.139	0.9575	22.343	0.8739	10.863	0.86
F8	11.535	0.9764	0.127	0.9761	21.065	0.9714	16.803	0.818
F9	10.886	0.9621	0.139	0.9468	25.315	0.9729	15.855	0.743

**Table 5:** In vivo skin irritation test.

Table	Mean Erythema ± SD	Mean Edema ± SD	Mean Combined ± SD
24	0.33 ± 0.58	0.00 ± 0.00	0.33 ± 0.58
48	0.33 ± 0.58	0.00 ± 0.00	0.33 ± 0.58
72	0.00 ± 0.00	0.00 ± 0.00	0.00 ± 0.00

Primary Irritation Index (PII): total combined scores = 2; number of observations = 3 rats × 3 timepoints = 9; PII = 2/9 = 0.22. interpretation: PII = 0.22 (PII < 0.5) classified as non-irritant/negligible irritation.

**Table 6:** Composition of nine formulations of nystatin-loaded novasomal gel.

Ingredients	F1	F2	F3	F4	F5	F6	F7	F8	F9
Nystatin (mg)	10	10	10	10	10	10	10	10	10
Span 60 (mg)	50	70	90	140	150	160	90	100	110
Oleic acid (mg)	5	10	20	-	-	-	5	10	15
Stearic acid (mg)	-	-	-	40	50	60	20	25	30
Cholesterol (mg)	30	40	50	50	60	70	60	60	60
Ethanol: Phosphate buffer (mL)	1:5	1:5	1:5	1:5	1:5	1:5	1:5	1:5	1:5
Novasomal Suspension (mL)	25	25	25	25	25	25	25	25	25
Carbopol (g)	20	20	20	20	20	20	20	20	20
H_2_O (mL)	50	50	50	50	50	50	50	50	50
Nystatin-loaded novasomal gel (g)	30	30	30	30	30	30	30	30	30

## Data Availability

The original contributions presented in the study are included in the article/Supplementary Materials; further inquiries can be directed to the corresponding author.

## Supplementary Materials

The following supporting information can be downloaded at: https://www.mdpi.com/article/10.3390/gels11100774/s1, Figure S1: Standard calibration curve of Nystatin; Table S1: Method validation parameters for Nystatin quantification; Table S2: Method validation parameters for Nystatin quantification.
