# Supplementary material for: Development and Evaluation of Nystatin-Loaded Novasomal Gel for the Treatment of Candida albicans Infection: In Vitro Microbiological and Skin Compatibility Study
Source: Gels. 2025 Sep 25;11(10):774. doi: 10.3390/gels11100774 (PMC12564917; doi:10.3390/gels11100774)
Supplement: Supplementary file 1 [file gels-11-00774-s001.zip › gels-3694013-supplementary.pdf]

Supplementary Information

Development and Evaluation of Nystatin-Loaded Novasomal Gel for the Treatment of *Candida albicans* Infection: In Vitro Microbiological and Skin Compatibility Study

Muhammad Abid Mustafa [1,*], Muhammad Fahad [1], Maryam Mughal [1], Namra Rasheed [1], Saad S. Alqahtani [2] and Muhammad Zahid Iqbal [2]

[1] Department of Pharmaceutics, Faculty of Pharmaceutical Sciences, Lahore University of Biological and Applied Sciences, Lahore, Pakistan
[2] Department of Clinical Pharmacy, College of Pharmacy, King Khalid University, Abha, 61421, Saudi Arabia

S1. Standard Calibration Curve

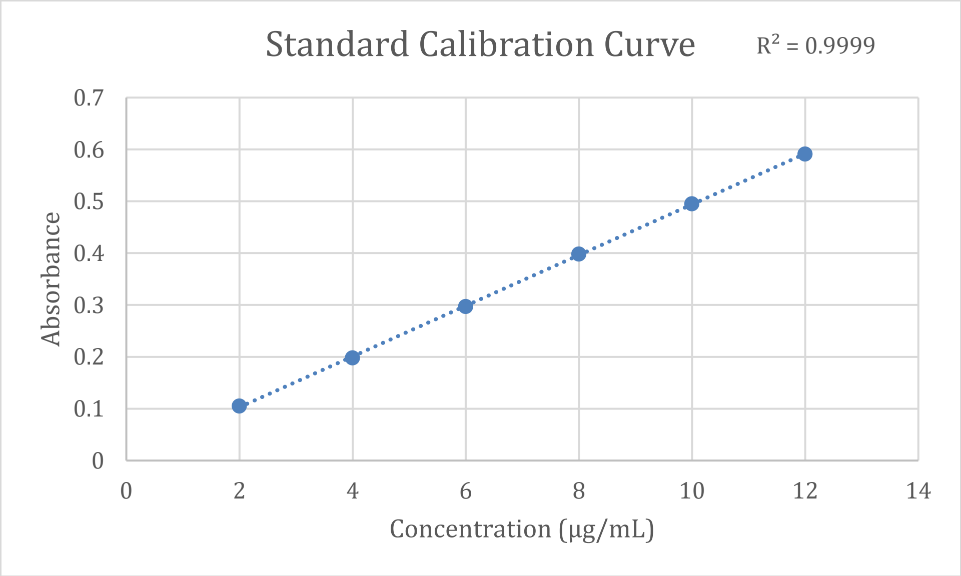

Figure S1. Standard calibration curve of Nystatin.

Table S1. Method validation parameters for Nystatin quantification.

**Parameter**	**Result**
Linearity range	2–12 μg/mL
Correlation coefficient	0.9999

Limit of Detection (LOD)	0.31 μg/mL
Limit of Quantification (LOQ)	0.95 μg/mL
Accuracy (% recovery)	98.7–101.2%
Precision (% RSD, n=3)	< 2%
Specificity	No interference from excipients
Repeatability	Confirmed

S2. In Vitro Drug Release Profile

The cumulative in vitro drug release profiles of Nystatin-loaded formulations (F1–F9) are presented in Table S2. Values are expressed as mean ± SD (n = 3).

Table S2. In vitro drug release profile of Nystatin-loaded formulations (mean ± SD, n=3).

Time (hours)	F1	F2	F3	F4	F5	F6	F7	F8	F9
0.5	9.23±0.41	7.89±0.17	10.2±0.79	12.1±0.82	12.91±0.48	13.3±0.81	8.99±0.05	9.93±0.76	10.23±0.29
1	20.02±0.19	15.99±0.43	14.31±0.92	22.52±0.07	22.92±0.83	23.42±0.09	14.65±0.57	19.25±0.48	20.79±0.65
2	29.04±0.72	34.65±0.94	24.92±0.07	36.17±0.71	37.21±0.34	38.72±0.45	23.63±0.82	27.55±0.29	30.64±0.71
3	38.99±0.81	50.76±0.05	34.22±0.23	39.83±0.38	43.34±0.11	44.32±0.73	27.69±0.93	40.27±0.95	39.46±0.69
4	54.23±0.65	63.91±0.44	45.44±0.12	60.22±0.28	56.5±0.19	58.61±0.31	39.27±0.20	50.63±0.11	49.39±0.83
5	70.66±0.20	75.34±0.76	59.13±0.23	65.31±0.67	66.27±0.43	67.54±0.62	49.42±0.56	57.37±0.27	58.26±0.90
6	85.29±0.87	80.21±0.13	70.21±0.73	79.42±0.87	80.63±0.72	76.36±0.12	60.13±0.84	65.84±0.45	64.56±0.76

7		83.66±0.09	84.14±0.54	83.11±0.33	89.65±0.39	80.63±0.49	67.05±0.32	70.69±0.67	76.34±0.59
8		86.43±0.51	86.72±0.82	89.69±0.89	90.99±0.88	85.93±0.23	75.24±0.64		83.38±0.06
9			88.54±0.93	90.65±0.44	91.42±0.63	87.35±0.81	77.43±0.39	85.12±0.77	87.21±0.61
10						89.62±0.34	89.65±0.23	88.32±0.31	89.05±0.47
11						90.32±0.93			
12						93.22±0.48			
